# Quantitative Analysis of *Rhodobacter sphaeroides* Storage Organelles via Cryo-Electron Tomography and Light Microscopy

**DOI:** 10.3390/biom14081006

**Published:** 2024-08-14

**Authors:** Daniel Parrell, Joseph Olson, Rachelle A. Lemke, Timothy J. Donohue, Elizabeth R. Wright

**Affiliations:** 1 Department of Biochemistry, University of Wisconsin—Madison, Madison, WI 53706, USA; dparrell@wisc.edu (D.P.); joeolson76@gmail.com (J.O.); 2 Wisconsin Energy Institute, University of Wisconsin—Madison, Madison, WI 53726, USA; rachelle.lemke@wisc.edu; 3 Great Lakes Bioenergy Research Center, University of Wisconsin—Madison, Madison, WI 53726, USA; 4 Department of Bacteriology, University of Wisconsin—Madison, Madison, WI 53706, USA; 5 Cryo-Electron Microscopy Research Center, Department of Biochemistry, University of Wisconsin—Madison, Madison, WI 53706, USA; 6 Midwest Center for Cryo-Electron Tomography, Department of Biochemistry, University of Wisconsin—Madison, Madison, WI 53706, USA; 7 Morgridge Institute for Research, Madison, WI 53715, USA

**Keywords:** polyhydroxybutyrate (PHB), polyphosphate (PP), cryo-electron tomography, cryo-electron microscopy, *Rhodobacter sphaeroides*, chloramphenicol, cell stress

## Abstract

Bacterial cytoplasmic organelles are diverse and serve many varied purposes. Here, we employed *Rhodobacter sphaeroides* to investigate the accumulation of carbon and inorganic phosphate in the storage organelles, polyhydroxybutyrate (PHB) and polyphosphate (PP), respectively. Using cryo-electron tomography (cryo-ET), these organelles were observed to increase in size and abundance when growth was arrested by chloramphenicol treatment. The accumulation of PHB and PP was quantified from three-dimensional (3D) segmentations in cryo-tomograms and the analysis of these 3D models. The quantification of PHB using both segmentation analysis and liquid chromatography and mass spectrometry (LCMS) each demonstrated an over 10- to 20-fold accumulation of PHB. The cytoplasmic location of PHB in cells was assessed with fluorescence light microscopy using a PhaP-mNeonGreen fusion-protein construct. The subcellular location and enumeration of these organelles were correlated by comparing the cryo-ET and fluorescence microscopy data. A potential link between PHB and PP localization and possible explanations for co-localization are discussed. Finally, the study of PHB and PP granules, and their accumulation, is discussed in the context of advancing fundamental knowledge about bacterial stress response, the study of renewable sources of bioplastics, and highly energetic compounds.

## 1. Introduction

Bacteria generally lack the membrane-bound organelles present in eukaryotic cells that enable them to manage discreet cellular functions associated with respiration, photosynthesis, the removal of toxic compounds, protein trafficking, and more. Indeed, the coordination of cellular processes is made more efficient by organelles, proteinaceous compartments, and phase-separated condensates [1]. A well-known and studied example of bacteria is the carboxysome, which are protein-coated microcompartments that contain enzymes for carbon dioxide fixation. The compact environment contained within the bacterial carboxysome is effective and enables bacteria to drive nearly 35% of global carbon fixation [2]. A significant advantage of compartmentalizing processes, either in membrane-bound organelles or via consolidation within a region of a cell, is the concentration and/or sequestration of biochemical reactions and their products. Substrates may be concentrated around the enzymes that convert them into valuable products. In kind, fragile intermediate products and enzymes may be protected from other ongoing activities in the cell’s cytoplasm. Microbial polyhydroxybutyrate (PHB) granules, like the carboxysome, also serve to compartmentalize carbon storage. The PHB polymer is stored in subcellular proteinaceous shelled granules [3,4,5]. The enzymes for PHB synthesis and depolymerization are located on the PHB granule surface where these processes are concentrated [6]. Additionally, protein modulators of PHB granule size are present on the PHB granule surface and provide a mechanism to regulate both the number and volume of granules within an individual cell [7].

Polyphosphate (PP) granules, also present in many bacterial species, serve as storage vessels for phase-separated inorganic phosphate. The formation of PP granules is catalyzed by the activity of polyphosphate kinase that converts ATP into PP and ADP [8]. The reversible polymerization coordinated by polyphosphate kinase allows PP synthesis to be regulated by the availability of cellular ATP. A possible advantage of this adaptation could be to couple PP to cellular processes that require larger energy reserves. PP granules appear to localize near several subcellular structures in bacteria. PP granules may associate with the nucleoid in *Agrobacter tumefaciens* and *Pseudomonas aeruginosa* [9,10]. PP granules seem to be essential for motility and may support flagellar motor function in *Helicobacter pylori* and *P. aeruginosa* [11,12], and PP granules are also present within dormant spores of *Acetonema longum* [13]. This synergy between the location of PP granules and these energy-intensive structures may relate to PP being an essential source of energy and its targeted position within a cell.

Recently, fluorescence microscopy and cryo-ET experiments have defined the subcellular localization of both PP and PHB granules in several bacterial species [4,9,14]. Here, we demonstrate the use of cryo-ET, fluorescence microscopy, and biochemical methods to quantify the role of PP and PHB granules in *Rba. sphaeroides* at steady-state and in response to treatment with chloramphenicol (Cm). Our results demonstrate the advantage of using cryo-ET to effectively study granules and other subcellular structures in intact single cells. Cm is a broad-spectrum antibiotic that blocks protein translation at the aminoacyl site of the ribosome, via binding to the 23s rRNA [15]. Halting cell growth with bacteriostatic antibiotics, such as Cm, can also disrupt metabolic activity [16,17]. Here, we used Cm treatment to arrest the growth of *Rba. sphaeroides* to define changes that occur to PHB and PP levels and localization. Cryo-ET data demonstrated that the Cm treatment of *Rba. sphaeroides* resulted in significant changes to the accumulation of both PHB and PP granules within cells. We also identified physical changes to individual PHB and PP granules in single cells, as well as the amount of material stored as related to total cell biomass. Understanding the relationship between antibiotic (Cm) treatment and PHB or PP accumulation will improve our understanding of the physiological response that bacteria have to antibiotics. In addition, bacterial storage compartments (e.g., PHB and PP granules) that are enriched in specific compounds may be desirable to engineer as bio-renewable building blocks for bioplastics, biofuels, and other industrial products [18].

## 2. Materials and Methods

### 2.1. Bacterial Strains and Media

*Rba. sphaeroides* strain 2.4.1 and a mutant lacking PHB production, *Rba. sphaeroides* Δ*RSP0382 (*Δ*phaC)* [19], were grown in Sistrom’s minimal medium with succinate as the carbon source [20]. The initial starter cultures were incubated at 30 °C, with shaking at 200 RPM in the dark for ~36 h, and were used as an inoculum for all experiments. In experiments with chloramphenicol (Cm) treatment, a 20 mL culture in 250 mL flasks was inoculated to a starting OD_600_ of 0.05, grown in the dark with shaking at 200 RPM to an OD_600_ of ~0.4 and split between two flasks with media prewarmed to 30 °C. Half of the culture was supplemented with 200 mg/mL of Cm while the other was left untreated. The cultures were returned to 30 °C and shaken at 200 RPM in the dark for 7 h. We note that these conditions allowed for the production of some intracytoplasmic membranes despite aerobic growth in the dark. 

A fusion between mNeonGreen and the PHB-associated protein PhaP was generated by creating a C-terminal fusion [21] between *phaP (RSP_0381)* and *mNeongreen* in the expression vector pIND5. mNeonGreen was amplified by PCR using primers mNeonGreen fwd and mNeonGreen rev; *phaP* was amplified by PCR using *phaP*-fwd and *phaP*-rev (Supplementary Table S1). These DNA fragments were incubated with NdeI-digested pIND5 at 50 °C for one hour in NEBuilder HiFi DNA Assembly Cloning Kit (New England Biolabs, Ipswich, MA, USA) for Gibson assembly and then transformed into *E. coli* DH5α. The resulting plasmid was pIND5-mNeonGreen-phaP. We confirmed this plasmid by whole plasmid sequencing (Plasmidsaurus, Eugene, OR, USA). The sequence confirmed plasmid was transformed into conjugative *E. coli* strain S17, which was then used for conjugation into *Rba. sphaeroides* 2.4.1 [22].

### 2.2. Sample Vitrification

Aliquots of the bacterial liquid culture were vitrified onto EM grids using a Vitrobot Mark IV (Thermo Fisher Scientific, Hillsboro, OR, USA). Culture samples (5 μL) were applied to Quantifoil Cu 200 R2/1 grids (Quantifoil Micro Tools GmbH, Großlöbichau, Germany) for one min, manually blotted with filter paper (Whatman no. 1) from the side of the grid, and reapplied 2 times; the final double-sided grid blotting was conducted using the Vitrobot Mark IV blot pads. During the third on-grid incubation, 4 μL of 10 nm gold fiducials (BSA Gold tracer, Electron Microscopy Sciences, Hatfield, PA, USA) was added to the 5 μL suspension of bacteria for one min. After blotting, the grids were automatically plunged into liquid ethane for vitrification. The frozen grids were stored under liquid nitrogen until imaged with the electron microscopes.

### 2.3. Cryo-Electron Microscopy and Cryo-Electron Tomography Data Collection

Cryo-EM images were collected on a Titan Krios G3i 300 kV transmission electron microscope (Thermo Fisher Scientific) equipped with a Gatan K3 direct-electron detector and BioQuantum energy filter operated with a 20 eV slit width (Gatan, Inc., Pleasanton, CA, USA). Tilt series were collected bidirectionally from 0° in 2° or 3° increments from −60° to +60° with a total dose of either ~120 e^−^/Å^2^ or ~45 e^−^/Å^2^ using SerialEM [23]. Tilt series images were collected in movie mode with a total of eight frames per movie (i.e., one image constituted eight frames), the pixel size was 4.603 Å (19,500×), and the nominal defocus used was between −4 and −10 μm.

### 2.4. Tomographic Reconstruction and Data Analysis

Images within the tilt series were motion-corrected post-acquisition using motioncorr2 [24]. Tomographic reconstructions of the motion-corrected tilt series were created using the R-weighted back projection scheme in Imod/Etomo (version 4.12.52) [25]. The final reconstructions were binned by two (final pixel size of 9.202 Å) and CTF-corrected. Tomograms were normalized using e2proc3d [26] and in Imod, a high-frequency cutoff equal to 0.07 was applied to reduce noise [25]. Tomograms were segmented using Eman2’s convolutional neural network (CNN)-based semi-automated cellular tomogram annotation workflow [26,27]. Eman2 models were converted to Imod models using imodauto, imodmesh, and imodsortsurf to create independent object models for each granule. The volumes of PHB and PP granules were individually determined from the output of imodinfo. For distance measurements between the two granule types, the mtk program in Imod was used to determine the closest distance between the surface of Imod objects in the model files. These data were processed using a combination of Microsoft Excel (Version 16.77.1, Microsoft, Redmond, WA, USA) and R Studio (Version 2023.09.1+494, PBC, Boston, MA, USA). For visualization purposes, the Eman2 (version 2.99) models for inner membrane (IM), outer membrane (OM), PP, and PHB were opened in ChimeraX (Version 1.6.1) along with the noise-reduced tomogram [28].

### 2.5. Dose Tolerance of Storage Granules

The relative dose tolerance of PHB and PP granules was determined by recording repeated exposures, e.g., a dose series, of individual cells to monitor the accumulated damage to cellular structures. Each exposure was at a pixel size of 4.603 Å (19,500×), a nominal defocus between −6 and −10 μm, delivered ~5 e^−^/Å^2^ dose to the cell, and 40 exposures were collected for a total applied dose of ~200 e^−^/Å^2^. Granules of each type were monitored over the course of the dose series for signs of beam-induced damage.

### 2.6. Extraction and Quantification of PHB

Extraction of PHB proceeded with an acidified methanol extraction followed by liquid chromatography–mass spectrometry (LC-MS). Samples of the *Rba. sphaeroides* cultures were normalized by adding media to the cell volume collected so that the OD_600_ was equivalent to 10 optical density units. The cells were then pelleted at 7000× *g* for 10 min and resuspended in 500 μL of Sistrom’s minimal medium. The samples were flash-frozen in liquid nitrogen and stored at −80 °C. The frozen samples were lyophilized overnight to freeze dry the bacterial biomass and were then weighed. The dry cells were then digested in 4 mL of acidified methanol containing 3% sulfuric acid and methyl benzoate at 0.025% (*V/V*) concentration at 105 °C overnight with occasional vortex mixing during the first hour. After digestion, the sample was cooled to room temperature, and undigested debris was pelleted via centrifugation at 4000× *g* for 5 min. Approximately 200 μL of the aqueous phase was collected and transferred to chromatography vials. Samples were analyzed by LC-MS as described below.

In total, 1 μL of each sample was analyzed on a Shimadzu Nextera XR system connected to a Simadzu LCMS-8045 mass spectrometry instrument (Shimadzu corporation, Kyoto, Japan) on a 2.6 μm PS C18 100Å column (Kinetex) with an acetonitrile and 0.2% formic acid gradient running at 0.4 mL/min at 50 °C. The column gradient program started at 100% acetonitrile and ramped to 14% with 0.2% formic acid from 0 to 6.5 min, the gradient then ramped to 95% with 0.2% formic acid from 6.5 to 8.5 min, and, finally, the gradient was lowered to 5% with 0.2% acetonitrile for the remainder of the run for 9.5–11 min. Positive ions were collected from 3 to 6 min and from 8 to 10.5 min for the PHB standard and the methyl benzoate internal standard, respectively. A commercial standard of methyl (S)-(+)3-hydroxybutyrate (Alfa Aesar, Haverhill, MA, USA, AAL1999606) was used for the quantification of ions; the precursor ion (118.9 m/z) and 3 product ions were targeted at 59, 87, and 101 m/z. An internal standard of methyl benzoate (Sigma 18344, Kawasaki, Japan) was run with the precursor ion at 136.95 m/z and the reference quantification ions at 59, 77, and 91 m/z. Chromatograms and mass spectra were analyzed using Shimadzu LabSolutions software version 5.91. A PHB standard (Alfa Aesar, Haverhill, MA, USA, AAL1999606) was used to produce a standard curve from 10–750 μg/mL and was run on LC-MS as described above. The LC-MS data were normalized to the internal standard and the mass of PHB was quantified relative to the total biomass. For the quantification of PHB content per cell, the mass of PHB was calculated relative to the number of cells in each sample after calculating the number of CFU/mL using dilution plating and enumeration.

### 2.7. Fluorescence Microscopy

Ambient temperature fluorescence microscopy on the same experimental cultures used for cryo-ET data collection was carried out on a Leica DMi8 epifluorescence microscope (Leica Microsystems, Wetzlar, Germany) equipped with a 100x oil immersion objective, a Texas red (Ex range = 540–580 nm, Em range = 592–668 nm) bandpass filter cube, a FITC (Ex range = 460–500 nm, Em range = 512–542 nm) bandpass filter cube, and a Hamamatsu C13440 digital camera. The dye, FM4-64 (AAT Bioquest, Sunnyvale, CA, USA), was used at a working concentration of 1 mg/mL to stain bacterial cell membranes. Cells were deposited onto 1.0% agarose pads on a glass slide and covered with a coverslip before imaging and immediately before aliquots were used for cryo-EM sample preparation.

## 3. Results

### 3.1. Tomographic Analysis of Rba. sphaeroides

**Figure 1:**
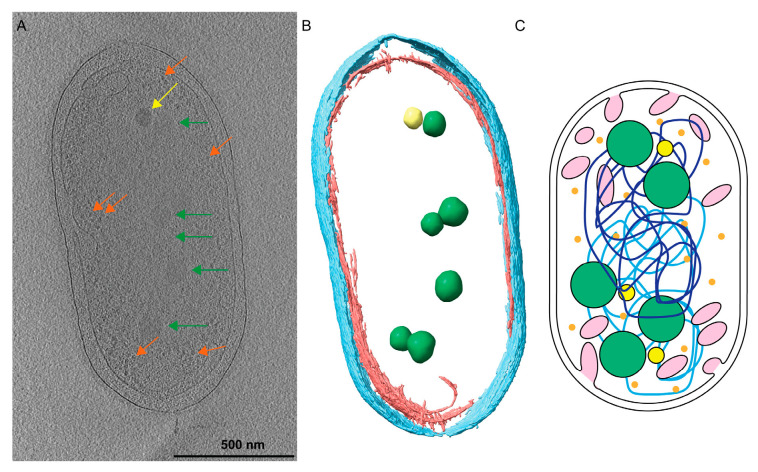
*Rhodobacter sphaeroides* cells contain many cytoplasmic structures. (**A**) A central slice from a tomogram of a *Rba. sphaeroides* cell shows the PHB granules (green arrows), PP granules (yellow arrows), and ribosomes (orange arrows) contained within the cytoplasm. A summed tomographic slice of 20 Z-planes is shown corresponding to a thickness of 184 Å. Scale bar is 500 nm. (**B**) Three-dimensional (3D) segmentation of the cell denotes PHB (green) and PP (yellow) granules contained within the inner (red) and outer (cyan) membranes. (**C**) A cartoon illustrating that *Rba. sphaeroides* cells contain intracytoplasmic membranes (pink), polyhydroxybutyrate (PHB, green spheres), polyphosphate (PP, yellow spheres), and ribosomes (orange), and the two chromosomes of *Rba. sphaeroides* (light- and dark-blue lines).

**Figure 2:**
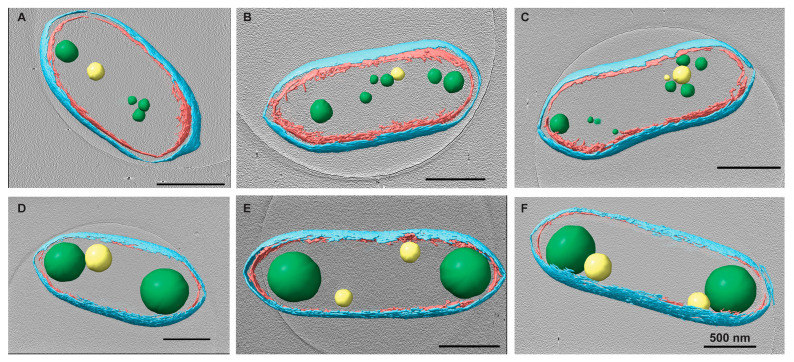
Changes in PHB granules upon chloramphenicol (Cm) treatment. *Rba. sphaeroides* cultures grown in Sistrom’s minimal medium with succinate as the carbon source. The culture was split in half, and one portion was untreated, i.e., no antibiotics (**A**–**C**) or treated with 200 μg/mL Cm (**D**–**F**). Central slices through tomographic reconstructions of cells in progressive stages of the cell cycle along with the presence of PHB granules and PP granules. Upon Cm treatment (**D**–**F**), PHB granules increased significantly in size compared to PHB granules in untreated cells (**A**–**C**). Segmentation models for each cell are overlaid onto the tomogram. The model depicts the inner (red) and outer (cyan) membranes as well as PHB (green) and PP (yellow) granules. Scale bars are 500 nm.

**Figure 3:**
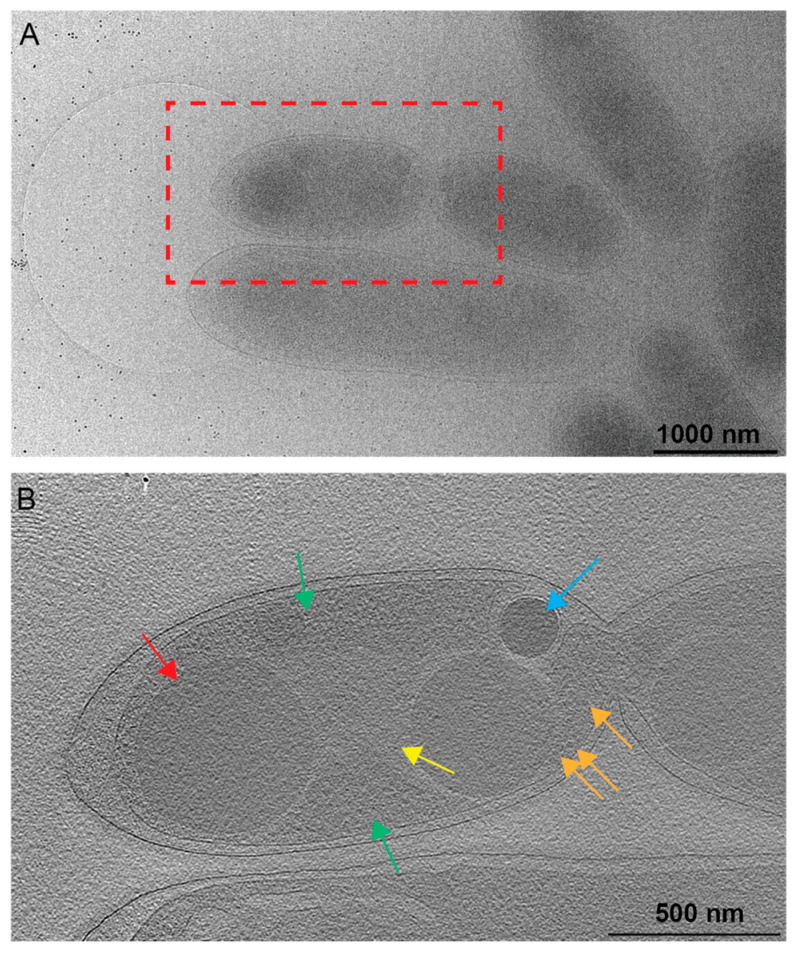
Nucleoid associations with PHB. (**A**) A 2D image of a *Rba. sphaeroides* cell just prior to cell division, with the red box indicating the location of the tomogram collected for panel (**B**). Scale bar is 1000 nm. (**B**) A central slice from the tomogram of the same cell as in panel A. Several cytoplasmic features are present. A less electron-dense region devoid of ribosomes contains the nucleoid (yellow arrow), while a more electron-dense region (green arrows) containing ribosomes (orange arrows) represents the cytoplasm. PHB granules are indicated by the red arrow and PP granules by the blue arrow. Scale bar is 500 nm.

We investigated the subcellular architecture of *Rba. sphaeroides* cells with cryo-electron tomography (cryo-ET). Three-dimensional (3D) reconstructions or tomograms of the cells were produced. Within the tomograms, subcellular structures were identified, including the outer and inner cytoplasmic membranes, ribosomes and storage granules, and nuclear material (Figure 1, Figure 2 and Figure 3, and Supplementary Figure S1). The two types of storage granules observed in *Rba. sphaeroides* were differentiated based on their size, electron density, and dose tolerance. The *Rba. sphaeroides* granules were examined and compared to *Caulobacter crescentus* granules that were examined by cryo-ET and whose composition was verified by electron energy loss spectroscopy (EELS) analysis [29]. In *Rba. sphaeroides*, PHB granules were larger but less electron dense than PP granules (Figure 2A–F and Supplementary Figure S1). In addition to their size difference (Figure 2 and Supplementary Figure S2), we showed that the PHB and PP granules differed in their sensitivity to electron dose (Supplementary Figure S3). The larger, less electron-dense PHB granules were more sensitive to electron dose and showed signs of beam-induced damage after as few as 45 to 50 e^−^/Å^2^ (Supplementary Figure S3). Conversely, the smaller, darker PP granules were able to withstand as much as 200 e^−^/Å^2^ without visible signs of radiation damage. These dose tests were completed on five replicate cells per treatment. We observed faint regions of clearing near granules in some cells; these regions presented filamentous patterns consistent with the nucleoid (Figure 3). These regions were also devoid of ribosomes, and the faint material appeared to contact and slightly surround PHB granules.

To validate the identities of the PHB and PP granules, we engineered and used a Δ*phaC* (Δ*RSP0382*) mutant strain of *Rba. sphaeroides*. By deleting *phaC*, which encodes poly-beta-hydroxybutyrate polymerase, the polymerization of PHB was halted and resulted in the absence of PHB granules [19,30]. Cryo-EM grids of this strain were prepared, and tilt series were collected for downstream tomographic reconstruction and analysis. The Δ*phaC* mutant did not produce the larger, less electron-dense PHB granules but did produce the smaller more electron-dense PP granules (Supplementary Figure S4A,C). 

### 3.2. Changes to PHB Granules Upon Chloramphenicol Treatment

**Figure 4:**
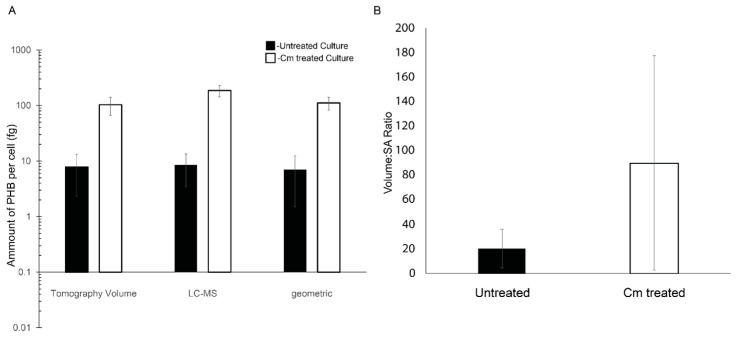
PHB granule content of *Rba. sphaeroides* cells. (**A**) A log-scale plot reporting accumulation of PHB calculated on a per-cell basis using segmentation models from 3D reconstructions (*n* = 22 for untreated cells and *n* = 20 Cm-treated cells), LC-MS data (*n* = 4 experiments) normalized to the number of cells in the culture, and by the calculation of PHB volume using cell diameters (*n* = 22 for untreated cells and *n* = 20 for Cm-treated cells) and the assumption of spherical PHB granules. Error bars represent the standard deviation of the mean. (**B**) A linear scale plot of the ratio of total PHB granule volume to PHB surface area per cell and averaged for all cells, untreated cells (black bar), and Cm-treated cells (white bar). Error bars represent the standard deviation of the mean. *n* = 22 for untreated cells and *n* = 20 for Cm-treated cells.

Given the roles of PHB and PP as carbon and energy stores, we asked if arresting cell growth would cause changes to granule accumulation and localization. We used chloramphenicol (Cm) to arrest growth and assess whether granule formation was altered due to the inhibition of translation. Untreated wild-type *Rba. sphaeroides* cells (22 tomograms of individual cells from 2 replicate experiments) contained ~7 PHB granules per cell. Cells from the same culture but treated with Cm (20 tomograms of individual cells from 2 replicate experiments) had ~1–3 PHB granules per cell. We measured the diameters of PHB granules and determined that the average diameter of PHB granules in untreated cells was ~179 nm (*n* = 158, ±55.6 nm), and the diameter of PHB granules from Cm-treated cells was nearly double at ~383 nm (*n* = 36, ±86.5 nm) (Supplementary Figure S2). This difference correlated with an approximately 9-fold increase in the volume of accumulated PHB in Cm-treated cells when granules were modeled as spherical objects in the tomograms (Figure 4A).

**Figure 5:**
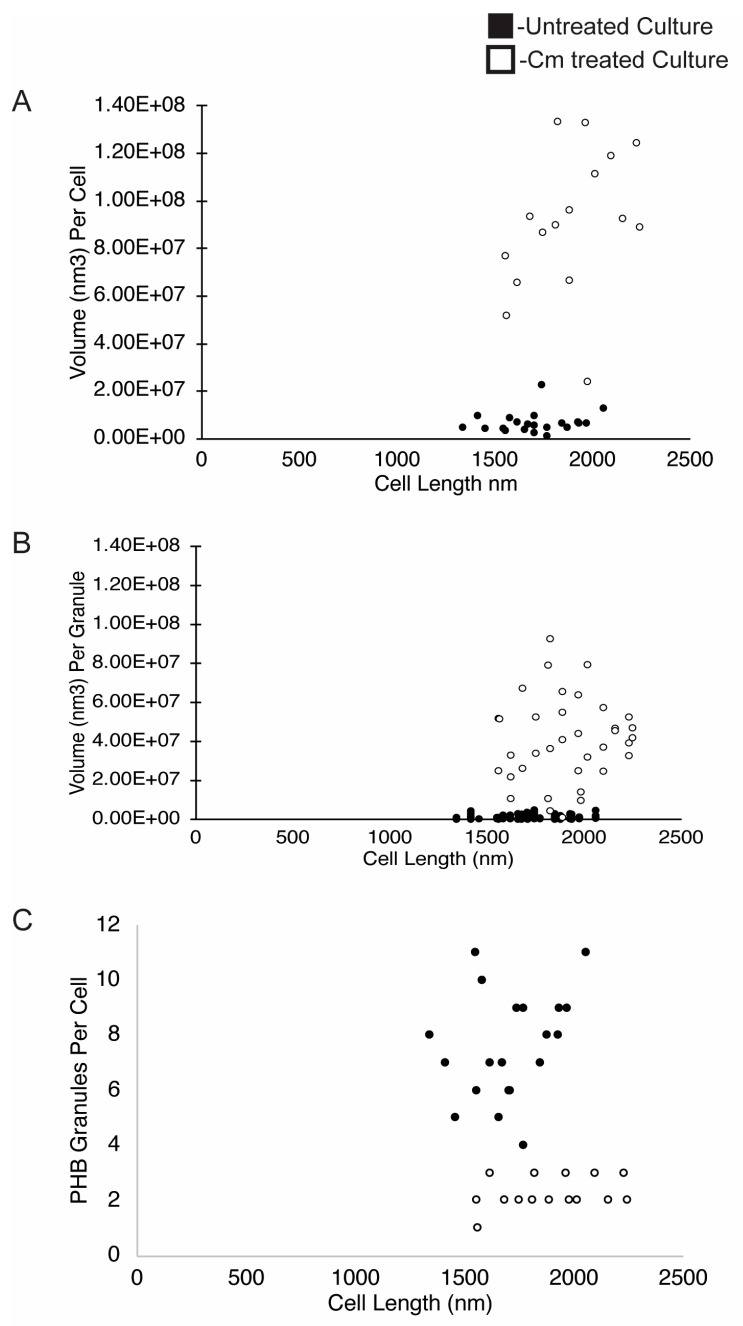
PHB volume, PHB volume per granule, and PHB number per cell. The volume of PHB was plotted on a per-cell (**A**) and per-granule (**B**) basis and is presented relative to the cell length (nm). (**C**) The number of PHB granules per cell was plotted relative to cell length (nm). Measurements are from untreated cells (black dots, *n* = 22 cells) or from Cm-treated cells (white dots, *n* = 20 cells).

To further define the amount of PHB per cell in the tomograms, the cells and PHB granules were segmented (Figure 2). The resulting models were used to measure the volume and surface area of PHB granules. The volume of PHB per cell was converted to the mass of PHB per cell using the density of PHB (1.170 g/cm^3^) [3]. This analysis revealed that Cm-treated cells had ~13-fold more PHB per cell, untreated cells had 7.8 femtograms (fg) (±5.5 fg) per cell, and Cm-treated cells had 103.3 fg (±36.6 fg) per cell. When the volumetric calculations were compared to the geometric data (Figure 4A), the surface area of PHB per cell increased ~3-fold from 3.2 × 10^5^ nm^2^ per untreated cell to 9.7 × 10^5^ nm^2^ per Cm-treated cell. This resulted in a ratio of PHB volume to surface area of 20.6 nm^3^/nm^2^ for untreated cells versus 90.3 nm^3^/nm^2^ for Cm-treated cells, a 4.5-fold difference (Figure 4B). However, as expected, this measure was variable due to the deviations in PHB volume within the few (1–3) granules per cell following Cm treatment (Figure 5A). When plotted individually, the volume of PHB per granule was consistently larger following Cm treatment (Figure 5B) as was the volume of PHB on a per-cell basis (Figure 5A). The volume of PHB per granule per cell did not correlate with cell length in untreated cells. However, in Cm-treated cells, the volume of PHB per cell increased as cells became longer (Figure 5A).

### 3.3. PHB Accumulation Measured by LC-MS

Cellular PHB was also quantified by LC-MS. This analysis showed that for 10 mg of dry cell mass, the PHB mass was ~130 µg for untreated cells and ~607 µg for Cm-treated cells. The quantity of PHB extracted was 1.2% of the total biomass of the untreated culture and increased to 6.0% of the total biomass of the Cm-treated culture. On a per-cell basis, in the untreated culture, there was approximately 8.5 femtograms (fg) (±5.0 fg) of PHB accumulated per cell, compared to 186 fg (±42 fg) of PHB per cell from Cm-treated cultures (Figure 4A). This represented a ~21-fold increase in PHB content when cells were in a growth-arrested state due to treatment with Cm. 

### 3.4. The Number and Localization of PHB Granules Is Altered by Cm Treatment

**Figure 6:**
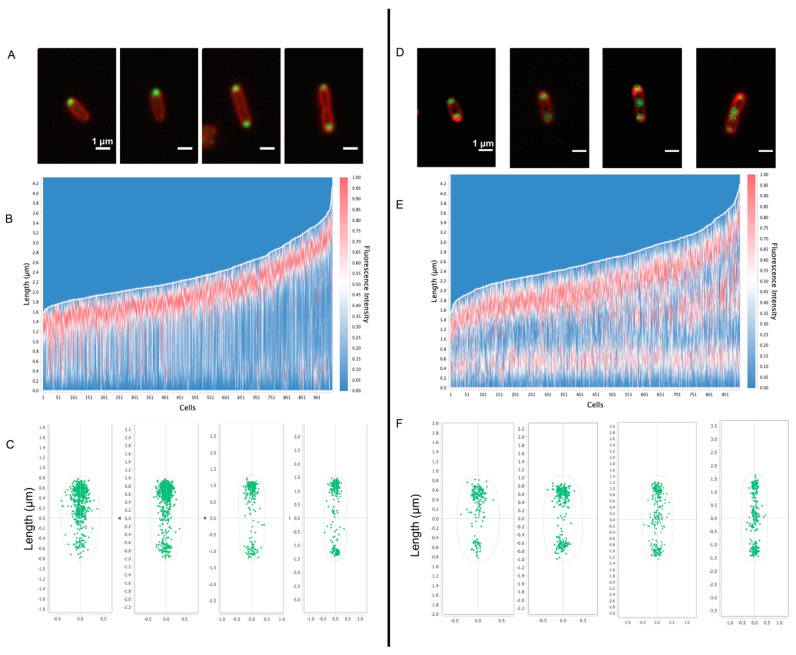
Fluorescence microscopy of PHB granules in *Rba. sphaeroides***.** For fluorescence visualization of PHB, the PHB-associated protein PhaP was fused to mNeonGreen. Panels (**A**–**C**) show data from untreated cells (*n* = 950 cells) and panels (**D**–**F**) show data from Cm-treated cells (*n* = 895 cells). Panels (**A**,**D**) are representative images of cells from the condition. mNeonGreen-PhaP fluorescence is shown in green and cell membranes, stained with FM 4–64, are depicted in red. Scale bars are 1 μm. Panels (**B**,**E**) represent a heatmap of mNeonGreen fluorescence plotted relative to cell length, with the brightest fluorescence oriented to the top pole. Panels (**C**,**F**) show the localization of fluorescent foci for cells from untreated and Cm-treated samples based on cell length.

As described above, wild-type *Rba. sphaeroides* cells contained, on average, 7 PHB granules per cell in untreated cultures, and Cm-treated cells contained ~1–3 PHB granules per cell. Despite this, the average volume of PHB per cell increased by ~13-fold when cells were in a growth-arrested state due to treatment with Cm. When the number of granules per cell was plotted relative to cell length (Figure 5C), the data showed that cells from the untreated culture had more granules per cell as cells lengthened. In samples of the Cm-treated culture, however, we consistently observed between 1 and 3 PHB granules per cell regardless of cell length (Figure 6).

To assess the presence and location of PHB granules in cells, the PHB intein protein (PhaP) [31] was labeled with a C-terminal mNeonGreen fusion (Figure 6). In untreated cells, PhaP fluorescence coalesced to the cell poles. Fluorescent foci at both poles were more frequent in cells that were longer and approaching cell division (Figure 6A). In contrast, the fluorescent foci were largely located at both poles in Cm-treated cells, with the presence of midcell foci in cells that were close to dividing (Figure 6D). The fluorescent patterns observed in this strain generally agree with the analysis of our tomograms in which PHB granules were often observed at the poles of untreated cells and were localized to the poles and midcell after Cm treatment (Figure 2).

We also used cryo-ET to assess PHB granule distribution within cells before and after Cm treatment and growth arrest. To test for differences, the nearest-neighbor distance between PHB granules, the diameters of PHB granules, and the cell dimensions were determined and plotted on a three-axis scatter plot (Supplementary Figure S5). We found that untreated cells had smaller granules with diameters of ~100 nm or less, and Cm-treated cells had larger granules of 300–500 nm in diameter. However, granule diameter did not appear to correlate with cell length in either growing or growth-arrested cells. The tomograms showed that PHB granules in untreated cells remained clustered together, while PHB granules were dispersed along the length of Cm-treated cells (Supplementary Figure S5). These results agreed with fluorescence microscopy images of PhaC-labeled cells in which PHB was present at a single pole in untreated cells and dispersed to both poles following Cm treatment (Figure 6).

### 3.5. PP Granules Accumulate upon Cm Treatment

**Figure 7:**
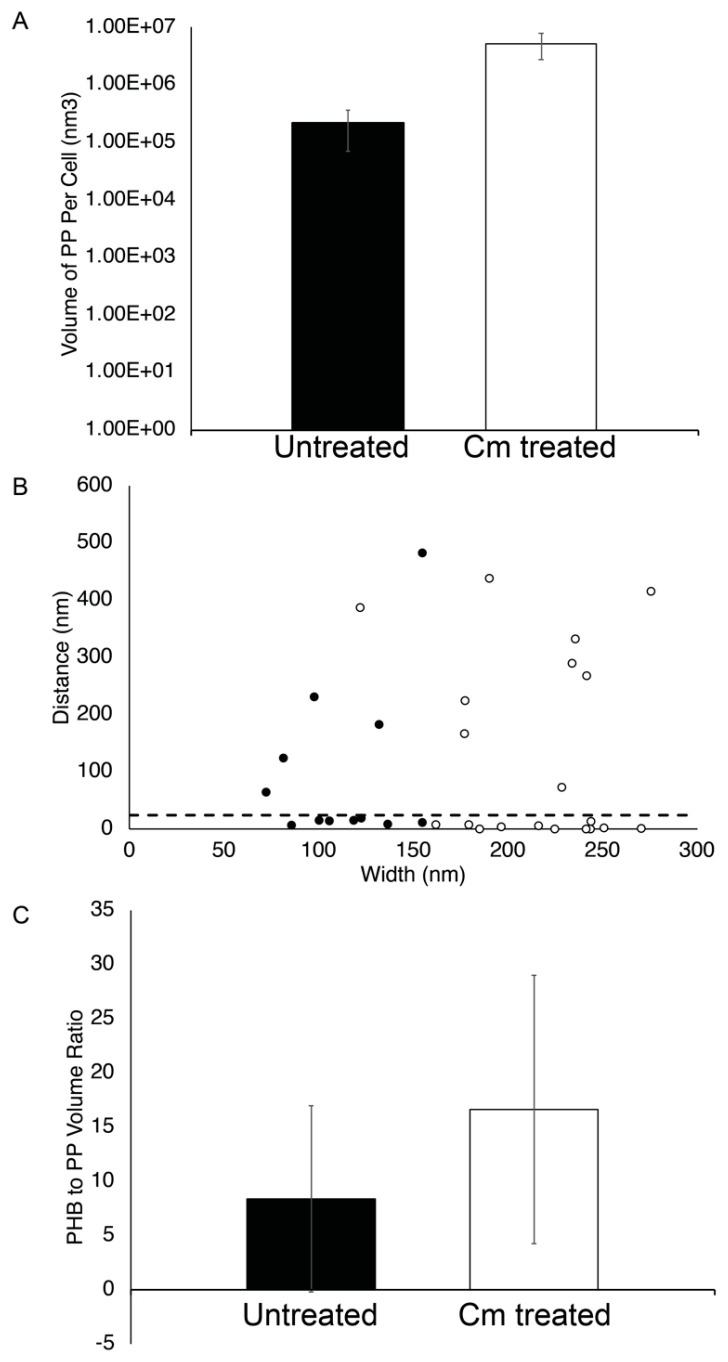
PP granule properties. (**A**) The volume of PP per cell was calculated using segmentation models and plotted for *Rba. sphaeroides* cells that were untreated (black bar, *n* = 23 granules in 22 cells) or Cm-treated (white bar, *n* = 26 granules in 20 cells). The y-axis is a logarithmic scale. (**B**) The distance between PP and PHB granules located at the same pole was measured for untreated and Cm-treated cells, plotted as a function of PP granule width. A dotted line indicates where 25 nm is located on the y-axis. (**C**) The ratio of PHB volume to PP volume per cell was calculated from segmentation modes and plotted for *Rba. sphaeroides* cells untreated (black bar, *n* = 23) or Cm-treated (white bar, *n* = 26).

We also analyzed the impact of Cm-mediated growth arrest on PP accumulation and localization. We found that the average diameter of PP granules was ~106 nm (±34 nm) in untreated cells versus 202 nm (±34 nm) after Cm treatment. Thus, we conclude that the size of PP granules in wild-type *Rba. sphaeroides* cells increased after Cm-mediated growth arrest. However, the number of PP granules per cell did not change significantly in growing or Cm-treated cells. In contrast, the volume of PP granules per cell, as measured by volume segmentation, was 8.5 × 10^5^ nm^3^ (±5.7 × 10^5^ nm^3^) in untreated cultures cells compared to 5.3 × 10^6^ nm^3^ (±4.2 × 10^4^ nm^3^) after growth arrest by Cm (Figure 7A), representing an ~6.2-fold increase in PP granule levels after Cm treatment.

### 3.6. Cytoplasmic Distribution of PHB and PP Granules

**Figure 8:**
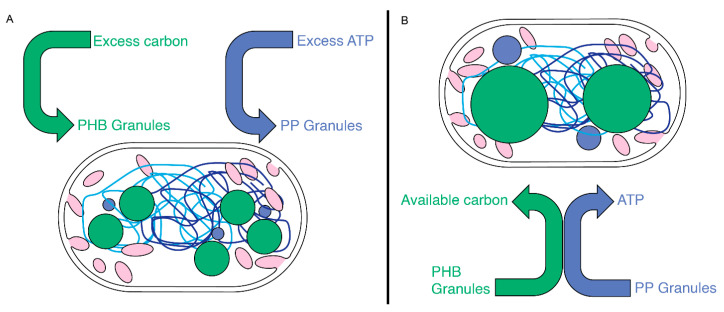
A model for PHB and PP generation and utilization in *Rba. sphaeroides*. (**A**) Upon experiencing excess nutrients, carbon, or ATP, *Rba. sphaeroides* cells accumulate carbon into PHB granules (green circles) and inorganic phosphate into PP granules (blue circles). (**B**) When PHB and PP stores are used, the physical proximity of PP and PHB granules may serve to couple the depolymerization of both granules with inorganic phosphate from PP feeding an ATP-requiring step in PHB depolymerization. The two chromosomes of *Rba. sphaeroides* (light- and dark-blue lines).

We also used the tomograms to measure the subcellular localization of PP and PHB granules. This analysis showed that approximately half (7 out of 12 granules) of the PP granules were within ~25 nm or less of PHB granules in untreated cells (Figure 7B). Following Cm treatment, PP granules were still closely associated with PHB granules with 11 out of 20 granules localizing within 25 nm of a PHB granule. Since there appeared to be little correlation between PP granule width and distance to PHB granules (Figure 7B), we asked if there was a relationship between the volume of PHB and the volume of PP per cell. We found that there was an approximately two-fold change in the ratio of PHB volume to PP volume in growth-arrested cells, although this measurement was highly variable. This suggested that despite the accumulation of both PHB and PP in Cm-treated cells (Figure 4A and Figure 8A), there was a tendency to accumulate more PHB than PP in cells under growth arrest (Figure 7C).

## 4. Discussion

### 4.1. PHB and PP Granules Respond to Cm Treatment by Accumulation

Bacterial cytoplasmic organelles separate and localize important or labile cellular processes from the rest of the cytoplasm and some of them do so without the use of membrane-bound compartments. PHB and PP granules are subcellular organelles that provide nutrient storage, localized access, and the concentration of their nutrients in the cytoplasm. In this study, we asked how PHB and PP granules are altered by growth arrest caused by the addition of Cm. We observed the difference between PHB and PP granules, as previously established in *C. crescentus.* The PP granules were more electron-dense and maintained a higher tolerance to electron beam irradiation, relative to the less dense PHB granules that exhibited electron beam sensitivity at approximately 45 to 50 e^−^/Å^2^. We used the cryo-ET data to generate 3D volumes of *Rba. sphaeroides* cells and volume segmentation to model the cell, PHB granules, and PP granules. We also used fluorescence microscopy to identify PHB granules and LC-MS to quantify PHB accumulation. These analyses were used to characterize the changes to the accumulation and localization of these membrane-less organelles that were associated with Cm treatment. Our results demonstrate an approximately 10–20-fold increase in PHB in Cm-treated cells. This increase in PHB in response to Cm suggests that PHB accumulation may be a mechanism that cells use to resist or cope with stressors [32,33,34,35,36,37]. In addition to the accumulation of PHB, following Cm treatment, we observed fewer granules per cell. In this case, the granules remained localized at the cell poles and were sometimes present at the midcell. We also observed a correlation between the locations of PHB and PP granules that implies there may be a metabolic or other advantage to this arrangement of these two energy-storage granules.

Unlike some cytoplasmic bacterial structures such as magnetosomes [38] or intracytoplasmic membranes [39], PHB and PP granules are not membrane-bound. This presents an interesting relationship between the synthesis and consumption of these resources. Membrane-bound organelles effectively compartmentalize reactions and substrates from the rest of the cytoplasm to sequester reactions from other processes [40]. Because PHB and PP granules lack membranes, these structures phase separate by forming a polymer. PP granules are a pure assemblage of inorganic phosphate polymers. PHB granules, on the other hand, are wrapped in a proteinaceous shell consisting of several granule-associate proteins [4,41]. These proteins play crucial roles in maintaining the size and number of granules in the cell [7]. Given that *Rba. sphaeroides* expresses many conserved homologs of these proteins, it is interesting to consider how Cm treatment affects the expression and/or regulation of these genes, promoting the differences we observe in granule size, number, and distribution. Our results demonstrate a dramatic change in not just the accumulation of PHB but also in the number and distribution of PHB granules in cells before and after growth arrest. The increase in PHB as a percent of the dry cell weight was nearly 6-fold following Cm treatment and reached ~6% of the cell dry weight in Cm-treated cells. In related organisms, such as *R. euthropha,* the percent of dry cell weight can be as high as 70% [42]. In addition to differences in PHB accumulation by bacterial species, it is important to consider how growth conditions impact PHB accumulation. For example, in the closely related bacterium *Rhodobacter capsulatus*, growth on acetate or caproate significantly increases PHB accumulation relative to other carbon sources [43]. Additionally, previous work in *Rba. sphaeroides* grown anaerobically in ammonium sulfate, succinate, and yeast extract (aSy) medium, supplemented with pyruvate, resulted in PHB accumulation up to ~40% of the dry cell weight [44]. This was significantly more than other carbon sources examined in this study. Similarly, the growth of *Rba. sphaeroides* under anaerobic conditions in SIS medium supplemented with several different organic substrates showed glucose to be the most optimal substrate for PHB production [30]. Additionally, the growth of *Rba. sphaeroides* during cycles of light and dark appears to promote PHB accumulation, suggesting a potentially fruitful cooperation between PHB and circadian cycles [45]. Understanding our results in the context of these other results highlights how a range of growth conditions can modulate, and even promote, PHB accumulation in *R. sphaeroides,* supporting the use of this organism as a microbial cell factory [46]. Knowing the capacity to modulate PHB accumulation in the context of these other findings may lead to synergy between PHB accumulation and the production of other compounds of interest.

### 4.2. PHB Localization

Previous cryo-EM work of PHB granules in *R. eutropha* demonstrated that granules are evenly distributed along the length of cells, with some bias toward the center [4]. Subsequent fluorescence microscopy and conventional TEM studies showed that while granules are dispersed throughout the cytoplasm, the distribution is not random [47]. A DNA-binding protein, PhaM, was shown to tether PHB to the nucleoid [6,47]. This is different from *Rba. sphaeroides* PHB granules that are restricted to the cell poles. PHB nucleoid association by PhaM is accomplished by proline and lysine residues present in a PAKKA motif [6]. A *R. eutropha phaM* mutant has increased granule size and loses localization with the nucleoid. Interestingly, *Rba. sphaeroides* does not have a *phaM* PAKKA homolog, and the pattern of subcellular localization is polar rather than distributed along the cell length. We do, however, observe apparent interactions between PHB granules and the nucleoid region in some cells (Figure 3). These results suggest that there may be connections between PHB and the nucleoid in *Rba. sphaeroides* like *R. eutropha*. However, the association mechanism is unknown. Additional research in *Rba. sphaeroides* is required to further define the mechanisms that govern PHB localization. This information will identify both the conserved and variable core processes of PHB physiology across different bacterial species.

PHB granules in growing *Rba. sphaeroides* cells are locally grouped in sets of 3–4 at the poles, or less often near the mid cell. Granules in these groups are largely spaced within 400 nm of each other (Supplementary Figure S5). PHB granules of Cm-treated cells become singular at the poles, with occasional pairs of granules (Figure 2). The distance between these granules, as a result, almost always approaches the length of a cell (Supplementary Figure S5). The observed local networks of PHB granules may be a mechanism by which the cell partitions PHB granules between cells in preparation for cellular division. In *R. eutropha*, mechanisms for PHB partitioning are driven by nucleoid-associating proteins on the PHB granule surface [47]. Our observations of PHB subcellular localization are supported by fluorescence microscopy that shows in untreated cells and PHB accumulating at one of the poles of untreated cells leading up to cell division and at a single pole in newly divided cells (Figure 6A–C). In Cm-treated cells, the localization of PHB is at both poles in Cm-treated cells with additional PHB accumulating at midcell in cells approaching division (Figure 6D–F). These results show that, like in *R. euthropha*, mechanisms for partitioning PHB granules exist.

### 4.3. PHB and PP Granule Associations

As described above, PHB and PP exist as distinct polymeric structures. The co-localization observed between PP and PHB granules suggests a functional synergy. The process of producing these polymers covalently sequesters inorganic phosphate and carbon in the cell into large relatively inaccessible structures. The coordinated placement of these granules within a cell means there is a localized source of energy and carbon for the cell to use. It is possible that the co-localization of PHB and PP granules is the result of their hydrophobic properties, with their association driven to support optimal associations within the cytoplasm. It is also interesting to consider whether the subcellular localization of storage granules may contribute to the optimization of cellular processes. Evidence of this kind of relationship exists in other species. The use of PP granules to power ATP-driven processes was documented with flagellar motors in *Helicobacter pylori* and *P. aeruginosa.* The loss of PP granules by deleting PpK severely affected cell motility and swimming [9,11]. Considering PHB metabolism provides some ideas for the cooperative role of PHB and PP (Figure 8). PHB granules are generated by the polymerization of 3-hydroxybuterate into PHB polymers via a 3-hydroxybutyrate-CoA intermediate, whose production requires the cofactor coenzyme-A [48]. Conversely, the depolymerization of PHB involves a coenzyme-A linked step that eventually leads to the production of free acetate by an ATP-driven reaction that recycles the coenzyme-A cofactor [48]. Since PP granules maintain ATP levels, it is possible that the physical association between PHB and PP granules seen in *M. gryphiswaldense*, or the clustering as we observed with *Rba. sphaeroides*, may serve to couple the depolymerization of PHB and PP granules (Figure 8). The coupled depolymerization of these granules would, thus, generate the ATP required to recycle coenzyme-A and generate free acetate in the cell. Whether these co-coordinating activities are occurring, or whether granules associate because of their hydrophobic properties will be an interesting topic of future work.

## 5. Conclusions

In this work, we showed that *Rba. sphaeroides* PHB and PP granules accumulate in response to growth arrest caused by Cm treatment. PHB and PP granules are excellent molecular models for studying the synthesis and dynamics of non-membrane-bound organelles in bacteria [21]. They generate large subcellular aggregates that are traceable by fluorescence microscopy and their 3D structures can be examined within intact cells by cryo-ET. In this work, we demonstrate the advantages and potential benefits of using cryo-ET synergistically with other analytical methods to study granules in intact single bacterial cells. We used cryo-ET to enumerate and quantify the properties of PHB and PP granules on a per-cell basis within vitrified whole cells. With these methods, we showed that both PHB and PP accumulate upon Cm treatment and that their size and number are changed. We also showed that PHB localization is polar in *Rba. sphaeroides*, with additional midcell localization following Cm treatments. We correlated measurements of PHB mass per cell by cryo-ET and LC-MS, showing that our cryo-ET results correspond well with standard methods for PHB quantification. PHB and PP granules and other bacterial membrane-less organelles represent unique biological systems to study microbial physiology and cellular mechanisms, but they also offer exciting potential as tools for industrial and pharmaceutical use [49]. As sources of carbon storage, PHB granules deliver potential raw materials useful for bioproducts that will reduce human reliance on non-renewable fuel sources [50,51]. The protein coats of PHB granules may also be potential platforms for localizing reactions. The compartmentalization of reactions, substrates, and intermediates within a cell offers the chance to more efficiently synthesize complex molecules of interest.

## Data Availability

The raw data supporting the conclusions of this article will be made available by the authors upon request.

## Supplementary Materials

The following supporting information can be downloaded at https://www.mdpi.com/article/10.3390/biom14081006/s1: Figure S1: PHB granules accumulate in growing cells upon Cm treatment.; Figure S2: PHB width increases upon Cm treatment.; Figure S3: Dose dependence of PHB (red arrow) and PP (blue arrow) granule accumulation after Cm treatment of *Rba. sphaeroides*; Figure S4: PP granules increase in size after Cm treatment; Figure S5: Scatter plot of PHB granule geometric coordinates within *Rba. sphaeroides* cells; Table S1: Primers used in this study.
