# Supplementary material for: Quantitative Analysis of Rhodobacter sphaeroides Storage Organelles via Cryo-Electron Tomography and Light Microscopy
Source: Biomolecules. 2024 Aug 14;14(8):1006. doi: 10.3390/biom14081006 (PMC11352279; doi:10.3390/biom14081006)
Supplement: Supplementary file 1 [file biomolecules-14-01006-s001.zip › biomolecules-3078749-supplementary.pdf]

Quantitative Analysis of *Rhodobacter sphaeroides* Storage Organelles via Cryo-Electron Tomography and Light Microscopy

Parrell et al.

SUPPLEMENTARY INFORMATION.

SUPPLEMENTARY FIGURES, LEGENDS FOR SUPPLEMENTARY FIGURES, AND

SUPPLEMENTARY TABLES.

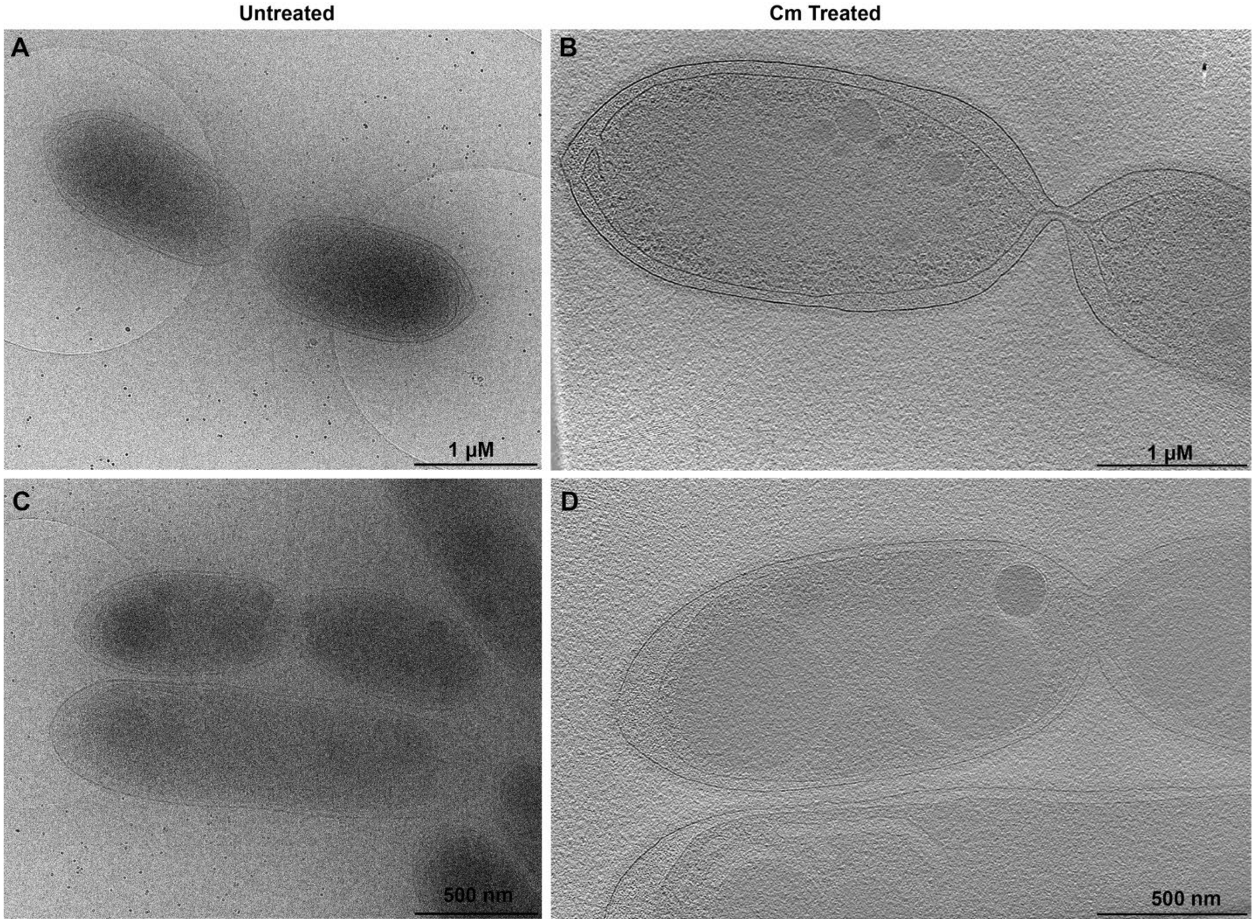

Supplementary Figure 1. PHB granules accumulate in growing cells upon Cm treatment. Cells undergoing cell division are shown at a nominal magnification of 4500x (A and C) capturing the entire cell. Scale bar is 1 µm. Central slices from the tomographic reconstructions of the same cells are presented in B and D showing PHB and PP granules localized within the cell along with the cleavage furrow. Scale bars are 500 nm. PHB granules of Cm-treated cells (C and D) demonstrate the accumulation of PHB under this condition. Cell in C and D is the same as in Figure 6.

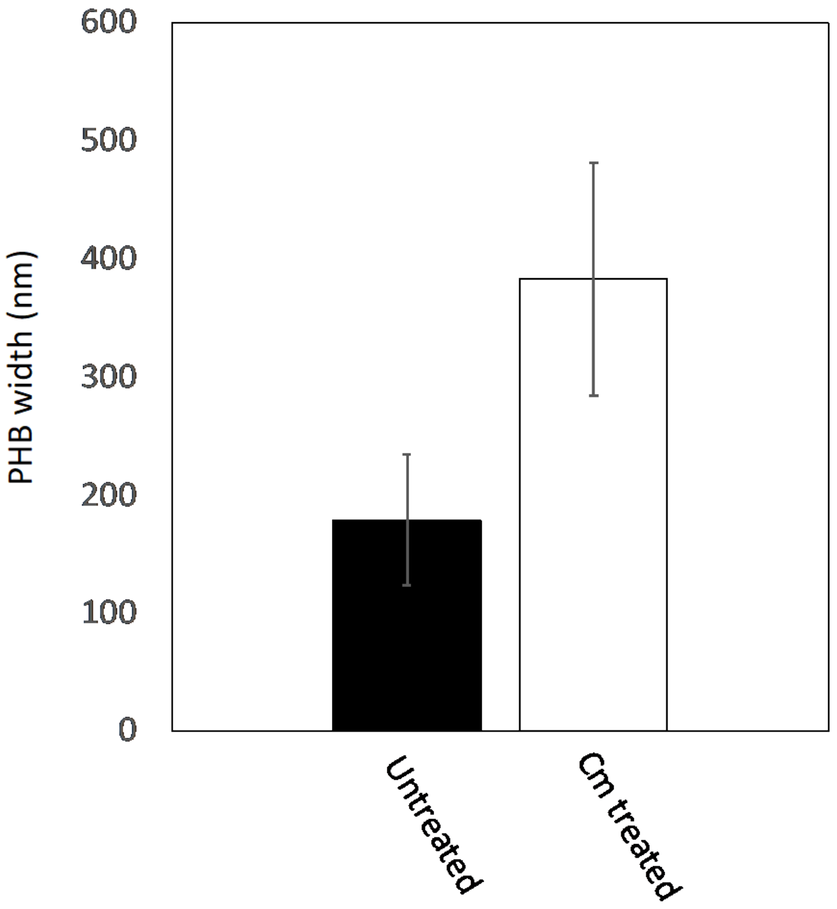

Supplementary Figure 2. PHB width increases upon Cm treatment. The width of PHB granules was measured and plotted for *Rba. sphaeroides* cells that were untreated (n=22 cells) and treated with Cm (n=20 cells). Error bars represent standard deviation of the mean.

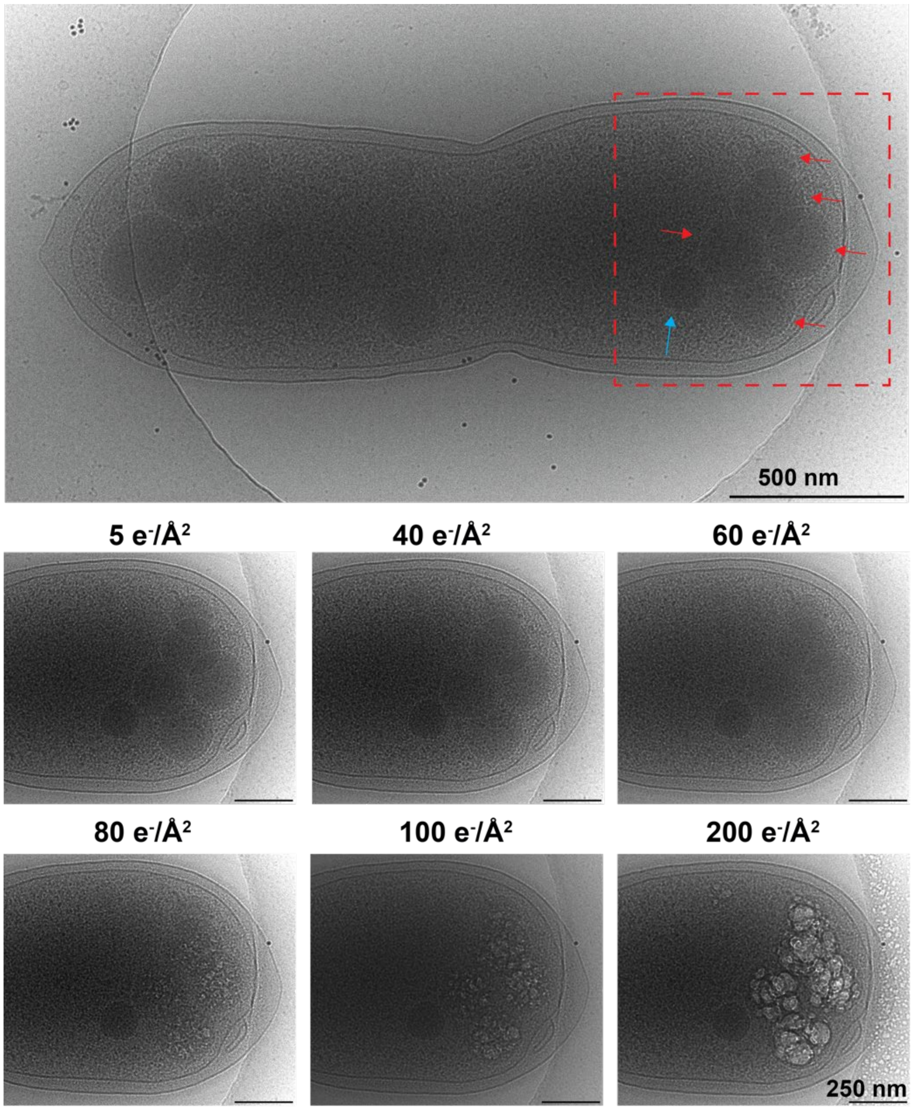

Supplementary Figure 3. Dose dependence of PHB (red arrow) and PP (blue arrow) granule accumulation after Cm treatment of *Rba. sphaeroides*. An intact cell was imaged using 2D cryo-EM. Repeated exposures were collected with a 5 $e^{-}/Å^{2}$ dose at the sample. Representative images are shown for cells treated with 5, 40, 60, 80, 100 and 200 $e^{-}/Å^{2}$. Arrows pointing to PP granules and PHB granules show the relative dose tolerance these structures. Scale bar is 500 nm for the top image, and 250 nm for the lower panels.

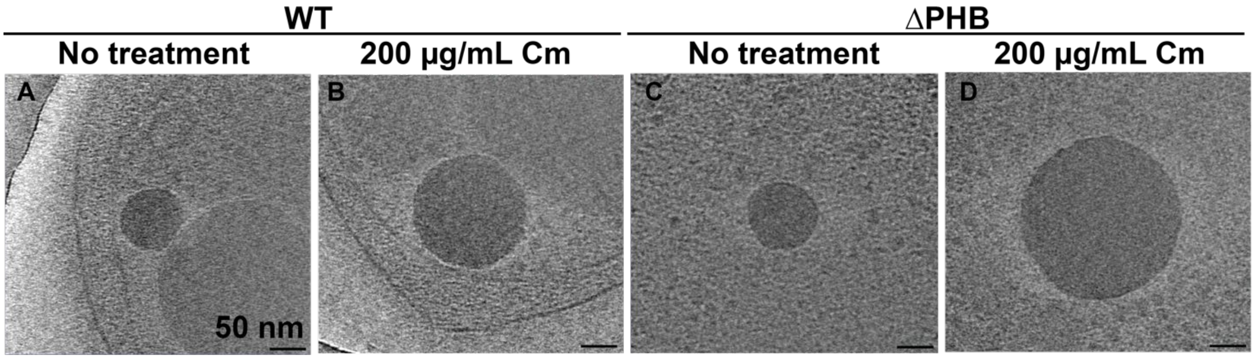

Supplementary Figure 4. PP granules increase in size after Cm treatment. (A and C) Untreated wild-type and *ΔphaC* cells maintain PP granules of ~75 nm in diameter. The *ΔphaC* mutant was used to eliminate the production of PHB granules. (B and D) PP granules significantly increase in size relative to granules of untreated wild-type and *ΔphaC* cells. Scale bars are 50 nm.

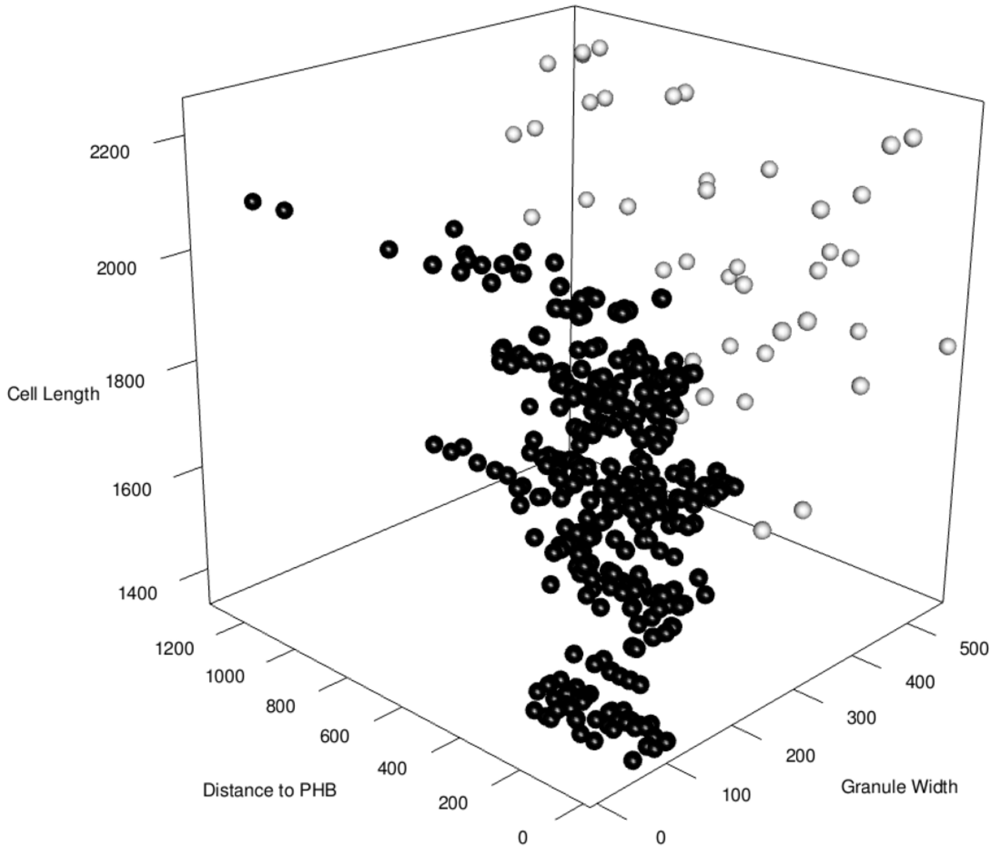

Supplementary Figure 5. Scatter plot of PHB granule geometric coordinates within *Rba. sphaeroides* cells. The distance between PHB granules, width of PHB granules and cell length plotted on a 3-axis scatter plot. Shown are the data from untreated cells (158 granules from 22 cells, black spheres) or Cm treated cells (36 granules from 20 cells, white spheres).

Primer	Sequence
***mNeonGreen*-for**	CGAAGGGTGGCGGAGGGTCTATGGTGAGCAAGGGCG
***mNeonGreen*-rev**	ATGAGATCTGGATCCTCCCATTACTTGTACAGCTCGTCCATGG
***phaP*-fwd**	ATTAAAGAGGAGAAATTACAATGACCAAGACCCCCGAC
***phaP*-rev**	CACCATAGACCCTCCGCCACCCTTCGCCGGAGCAGTG

Supplementary Table S1. Primers used in this study.
